# Supplementary material for: Viral-Mediated Optogenetic Stimulation of Peripheral Motor Nerves in Non-human Primates
Source: Front Neurosci. 2019 Jul 31;13:759. doi: 10.3389/fnins.2019.00759 (PMC6684788; doi:10.3389/fnins.2019.00759)
Supplement: Supplementary file 1 [file Table_1.DOCX]

Supplementary Material

# Supplementary Data:

**Supplementary Movie 1. Optically stimulated muscle contractions.** The movie clip demonstrates visible contractions of the right tibialis anterior muscle of Monkey M following viral transduction of ChR2 and optical stimulation of the corresponding deep peroneal nerve. The exposed nerve is stimulated with an optical fiber coupled to a 472 nm laser while electromyographic activity is recorded. Optical pulses are delivered at 2.5, 5, and 10 Hz, resulting in a fused tetanic contraction at 10 Hz followed by relaxation of the muscle after stimulation ceases.


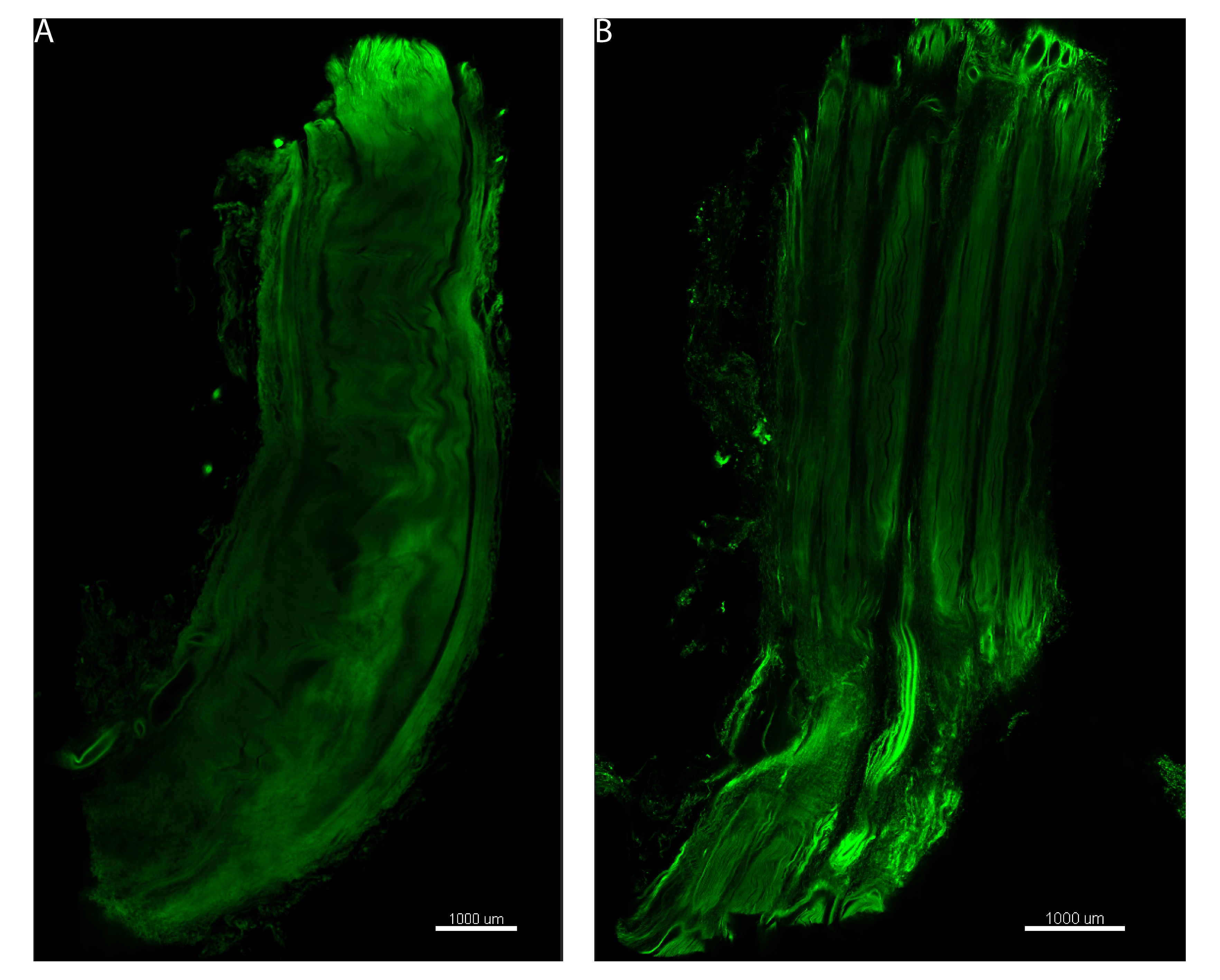


**Supplementary Figure S1.** **Whole sample imaging of nerves following loss of optical sensitivity.** Nerves that were previously sensitive to optical stimulation but demonstrated insensitivity to optical stimulation by the time of the terminal perfusion experiment were cleared using a PEGASOS protocol and imaged for native eYFP fluorescence using a ribbon scanning confocal microscope as described in Section 2.5 of the main text. A) Virus targeted branch of the median nerve innervating the right pronator teres muscle of Monkey O, and B) the deep peroneal nerve innervating the virus injected right tibialis anterior muscle of Monkey P. Neither nerve shows overt fluorescence in the axonal compartments compared to background signal. Bright sections of the top portion of the nerve in (A) likely correspond to tissue edge related artifacts, while bright portions in the lower half of (B) are associated with autofluorescence of fascia/connective tissue.
